# Supplementary figures and images for: Schistosoma haematobium infection is associated with oncogenic gene expression in Cervical Mucosa, with enhanced effects following treatment: A pilot study
Source: PLoS Negl Trop Dis. 2025 Nov 21;19(11):e0013569. doi: 10.1371/journal.pntd.0013569 (PMC12637897; doi:10.1371/journal.pntd.0013569)

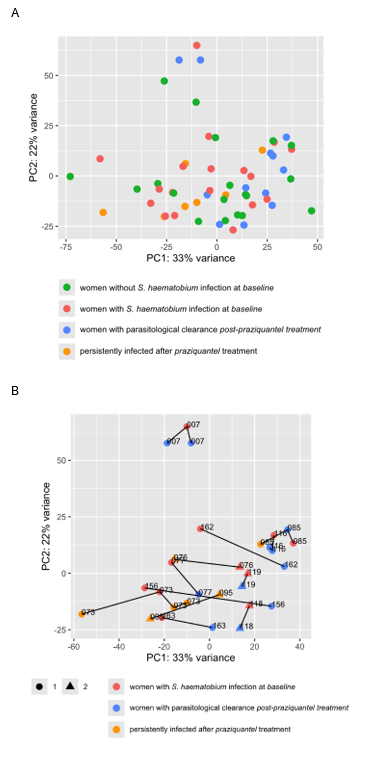

Supplement: S1 Fig — (TIF) [file pntd.0013569.s001.tif]
