# Supplementary material for: Schistosoma haematobium infection is associated with oncogenic gene expression in Cervical Mucosa, with enhanced effects following treatment: A pilot study
Source: PLoS Negl Trop Dis. 2025 Nov 21;19(11):e0013569. doi: 10.1371/journal.pntd.0013569 (PMC12637897; doi:10.1371/journal.pntd.0013569)
Supplement: S1 Table — (DOCX) [file pntd.0013569.s002.docx]

**Supplemental Table 1**. Top 9 differentially expressed genes between women with and without *S. haematobium* infection.

| **Gene name** | **NCBI Gene ID** | **Gene function** | **Disease associations and references** | **Log2 Fold Change and p-value** |
| --- | --- | --- | --- | --- |
| BLK proto-oncogene, Src family tyrosine kinase | BLK | Nonreceptor tyrosine-kinase of the src family of proto-oncogenes involved in cell proliferation and differentiation | T cell malignancies [1] | 1.4, p=0.003 by DESeq2, 1.2, p=0.04 by Limma |
| Collagen type VI alpha 4 pseudogene 2 | COL6A4P2 | Regulates structural roles and reported to influence cell migration via fibronectin-dependent means | Osteoarthritis [2] | 1.4, p=0.001 by DESeq2, 1.2, p=0.02 by Limma |
| ENSG00000260673 (novel transcript) | N/A | No data | N/A | -1.9, p=0.002 by DESeq2, -1.5, p=0.02 by Limma |
| ENSG00000275902 (novel transcript) | N/A | No data | N/A | 1.02, p=0.007 by DESeq2, 1.4, 0.001 by Limma |
| ENSG00000281195 (novel transcript) | N/A | No data | N/A | -1.3, p= 0.002 by DESeq2, -1.4, p=0.005 by Limma |
| Long Intergenic Non-Protein Coding RNA 2084 | LINC02084 | Prognostic maker for head and neck cancers and colon cancers | Head neck cancers [3] | 1.5, p=0.002 by DESeq2, -1.9, p=0.03 by Limma |
| Sterile alpha motif domain containing 3 | SAMD3 | Regulates cellular processes, including cell growth and proliferation, cell migration, and apoptosis | Aortic aneurysms and dissections with early on osteoarthritis [4] | 1.2, p=0.004 by DESeq2, 1, p=0.01 by Limma |
| Trichohyalin | TCHH | The protein encoded forms crosslinked complexes with itself and keratin intermediate filaments. | Gastric cancer [5] and liver metastasis in colorectal cancer [6] | -1.2, p=0.01 by DESeq2, -1.2, p=0.02 by Limma |
| TCL1 family AKT coactivator A | TCL1A | Coactivator of the serine threonine kinase AKT and through other interactions favoring cell survival, growth, and proliferation | T cell leukemia [7] | 2, p=5.05 x10^-4^ by DESeq2, 1.5, p=0.05 by Limma |

[1] Petersen DL, Krejsgaard T, Berthelsen J, Fredholm S, Willerslev-Olsen A, Sibbesen NA, et al. B-lymphoid tyrosine kinase (Blk) is an oncogene and a potential target for therapy with dasatinib in cutaneous T-cell lymphoma (CTCL). Leukemia 2014;28:2109–12. https://doi.org/10.1038/leu.2014.192.

[2] Nakajima M, Miyamoto Y, Ikegawa S. Cloning and characterization of the osteoarthritis-associated gene DVWA. J Bone Miner Metab 2011;29:300–8. https://doi.org/10.1007/s00774-010-0230-z.

[3] Deng H, Wei Z, Du J, Shen Z, Zhou C. Predicting the prognosis, immune response, and immunotherapy in head and neck squamous cell carcinoma using a novel risk model based on anoikis-related lncRNAs. Eur J Med Res 2023;28:548. https://doi.org/10.1186/s40001-023-01521-9.

[4] van de Laar IMBH, Oldenburg RA, Pals G, Roos-Hesselink JW, de Graaf BM, Verhagen JMA, et al. Mutations in SMAD3 cause a syndromic form of aortic aneurysms and dissections with early-onset osteoarthritis. Nat Genet 2011;43:121–6. https://doi.org/10.1038/ng.744.

[5] Yu F, Zhao L, Chu S. TCHH as a Novel Prognostic Biomarker for Patients with Gastric Cancer by Bioinformatics Analysis. Clin Exp Gastroenterol 2024;17:61–74. https://doi.org/10.2147/CEG.S451676.

[6] Chen A, Liu T, Bu D, Zhu J, Wang X, Pan Y, et al. Methylome profiling identifies TCHH methylation in CfDNA as a noninvasive marker of liver metastasis in colorectal cancer. FASEB J 2021;35:e21720. https://doi.org/10.1096/fj.202100266R.

[7] Virgilio L, Narducci MG, Isobe M, Billips LG, Cooper MD, Croce CM, et al. Identification of the TCL1 gene involved in T-cell malignancies. Proc Natl Acad Sci U S A 1994;91:12530–4. https://doi.org/10.1073/pnas.91.26.12530.

[8] Tang X, Xue D, Zhang T, Nilsson-Payant B, Carrau L, Duan X, et al. A multi-organoid platform identifies CIART as a key factor for SARS-CoV-2 infection. Nat Cell Biol 2023;25:381–9. https://doi.org/10.1038/s41556-023-01095-y.
