# Supplementary material for: Schistosoma haematobium infection is associated with oncogenic gene expression in Cervical Mucosa, with enhanced effects following treatment: A pilot study
Source: PLoS Negl Trop Dis. 2025 Nov 21;19(11):e0013569. doi: 10.1371/journal.pntd.0013569 (PMC12637897; doi:10.1371/journal.pntd.0013569)
Supplement: S2 Table — (DOCX) [file pntd.0013569.s003.docx]

**Supplemental Table 2.** Top 9 differentially expressed genes between women with parasitological clearance post-praziquantel versus women with baseline *S. haematobium* infection.

| **Gene name** | **NCBI**  **Gene ID** | **Gene function** | **Disease associations and references** | **Log2 Fold Change** |
| --- | --- | --- | --- | --- |
| Circadian associated repressor of transcription | CIART | Involved in circadian rhythm | SARS-CoV2 infection [1] | 1.9 p=1.94 x10^-5^ by DESeq2, 1.8, p=0.001 by Limma |
| C-X-C motif chemokine ligand 14 | CXCL14 | Encodes secreted proteins involved in immunoregulatory and inflammatory processes | Downregulated in HPV-positive head/neck and cervical cancers [2] | -1.6, p=3.48x10^-4^  by DESeq2,  -1.6, p=0.001 by Limma |
| ENSG00000280149 | N/A | No data available | N/A | -0.9 p=3.38 x 10^-3^  by DESeq2,  -1.4, p=4.90 x 10^-4^  by Limma |
| Long intergenic non-protein coding RNA 592 | LINC00592 | Proliferation, migration, and epithelial-mesenchymal transitions | Bladder [3] and breast cancer [4] | 1.3, p=0.01 by DESeq2, 2.1, p=3.37 x 10^-4^  by Limma |
| Lymphocyte antigen 6 family member K | LY6K | Involved in binding activity of sperm to zona pellucida | Oral squamous cell carcinoma [5] | 1.5, p=0.003 by DESeq2, 1.6, p=0.002 by Limma |
| Nuclear receptor subfamily 1 group D member 1 | NR1D1 | Negatively regulates core clock protein expression and may also modulate genes involved in metabolic, inflammatory, and cardiovascular processes | Bladder cancer [6] | 1.8, p=2.03 x 10^-4^ by DESeq2, 1.8, p=0.001 by Limma |
| NACHT and WD repeat domain containing 2 | NWD2 | Signal transduction for specific neuronal circuits | High grade lung cancer [7] | 1.3, p=0.02 by DESeq2, 1.9, p=0.001 by Limma |
| RPP38 divergent transcript | RPP38-DT | Integral component of membranes. | None reported. | -2.2, p=1.56 x 10^-4^ by DESeq2,  -1.8, p=6.70 x 10^-4^  by Limma |
| Transmembrane channel like 1 | TMC1 | Encode transmembrane proteins with unknown specific function | Hearing loss [8] | 1.3, p=0.005 by DESeq2, 1.6, p=0.002 by Limma |

[1] Tang X, Xue D, Zhang T, Nilsson-Payant B, Carrau L, Duan X, et al. A multi-organoid platform identifies CIART as a key factor for SARS-CoV-2 infection. Nat Cell Biol 2023;25:381–9. https://doi.org/10.1038/s41556-023-01095-y.

[2] Cicchini L, Westrich J, Xu T, Vermeer D, Berger J, Clambey E, et al. Suppression of Antitumor Immune Responses by Human Papillomavirus through Epigenetic Downregulation of CXCL14. mBio 2016;7:e00270-16. https://doi.org/10.1128/mBio.00270-16.

[3] Wu P, Zhang Z, Yuan Y, Zhang C, Zhang G, Xue L, et al. A tumor immune microenvironment-related integrated signature can predict the pathological response and prognosis of esophageal squamous cell carcinoma following neoadjuvant chemoradiotherapy: A multicenter study in China. Int J Surg 2022;107:106960. https://doi.org/10.1016/j.ijsu.2022.106960.

[4] Wu T, Li N, Wu X, Du Y, Tang Z. LncRNA LINC00592 mediates the promoter methylation of WIF1 to promote the development of bladder cancer. Open Med (Wars) 2023;18:20230788. https://doi.org/10.1515/med-2023-0788.

[5] Xu C, Gong R, Yang H. Upregulation of LY6K induced by FTO-mediated demethylation promotes the tumorigenesis and metastasis of oral squamous cell carcinoma via CAV-1-mediated ERK1/2 signaling activation. Histol Histopathol 2024:18725. https://doi.org/10.14670/HH-18-725.

[6] Yang Y, Bai Y, Wang X, Guo Y, Yu Z, Feng D, et al. Clock gene NR1D1 might be a novel target for the treatment of bladder cancer. Urol Oncol 2023;41:327.e9-327.e18. https://doi.org/10.1016/j.urolonc.2023.04.021.

[7] Ito Y, Usui G, Seki M, Fukuyo M, Matsusaka K, Hoshii T, et al. Association of frequent hypermethylation with high grade histological subtype in lung adenocarcinoma. Cancer Sci 2023;114:3003–13. https://doi.org/10.1111/cas.15817.

[8] Nishio Y, Usami S. Prevalence and clinical features of autosomal dominant and recessive TMC1-associated hearing loss. Hum Genet 2022;141:929–37. https://doi.org/10.1007/s00439-021-02364-2.
