# Supplementary material for: Schistosoma haematobium infection is associated with oncogenic gene expression in Cervical Mucosa, with enhanced effects following treatment: A pilot study
Source: PLoS Negl Trop Dis. 2025 Nov 21;19(11):e0013569. doi: 10.1371/journal.pntd.0013569 (PMC12637897; doi:10.1371/journal.pntd.0013569)
Supplement: S3 Table — (DOCX) [file pntd.0013569.s004.docx]

**Supplemental Table 3**. Overview of the top canonical pathways identified by Ingenuity Pathway Analysis.

| **Comparator** | **Top Canonical Pathway identified** | **Genes in Pathway, No.** | **Genes changed in Pathway, No. (%)** | **Transcripts^a^ in Pathway and Predicted Effects (fold change; P Value)** |
| --- | --- | --- | --- | --- |
| Women with *S. haematobium* infection versus without | None | N/A | N/A | N/A |
| Women with parasitological clearance post-praziquantel versus women with baseline *S. haematobium* infection | Sertoli Cell-Germ Cell Junction Signaling Pathway | 236 | 20 (8.5%) | **Predicted to be activated in participants with parasitological clearance:**  Role in the immune system:  transforming growth factor beta 2 (1.86; 5.03x 10 ^-7^)  mitogen-activated protein kinase 8 (1.014; 1.29 x10^-5^)  interleukin 1 receptor like 1 (2.237; 2.92 x10^-4^)  Role in oncogenesis:  RAS like proto-oncogene A (0.839; 7.84 x10^-5^)  RAP2B, member of RAS oncogene family (0.881; 2.28 x10^-4^)  NRAS proto-oncogene, GTPase (0.557; 1.14 x 10^-3^)  Other:  mitogen-activated protein kinase kinase kinase 13 (0.764; 3.91 x10^-5^)  integrin subunit alpha 2 (1.268; 2.36 x 10^-4^)  mitogen-activated protein kinase kinase kinase 9 (1.047; 1.38x^-3^)  **Predicted to be inhibited in participants with parasitological clearance:**  Role in epithelial barrier or cytoskeleton integrity:  afadin adherens junction formation factor (1.069;4.31 x10^-5^)  claudin 1 (1.551; 2.86E-04)  claudin 16 (1.39; 0.5.71 x 10^-4^)  nectin cell adhesion molecule 2 (0.505; 7.06 x 10^-4^)  WASP like actin nucleation promoting factor (0.992; 7.63 x 10^-4^)  tight junction protein 1 (0.911; 7.80 x 10^-4^)  CXADR Ig-like cell adhesion molecule (0.752; 9.17 x 10^-4^)  laminin subunit beta 3 (0.71; 1.61 x10^-3^)  Other:  protein phosphatase 2 regulatory subunit B'epsilon (0.324; 1.41 x 10^-3^)  sorbin and SH3 domain containing 1(1.047; 1.39 x 10^-3^)  protein phosphatase 2 regulatory subunit Bgamma (1.398; 7.57 x 10^-4^) |
| Women with parasitological clearance post-praziquantel versus women without *S. haematobium* infection at baseline | Activin Inhibin Signaling Pathway | 211 | 11 (5.2 %) | **Predicted to be activated in participants who cleared the infection:**  Role in the immune system:  immunoglobulin heavy constant gamma 1 (G1m marker) (-2.654; 0.037)  immunoglobulin heavy constant gamma 2 (G2m marker) (-2.435; 0.036)  immunoglobulin heavy constant gamma 4 (G4m marker) (-2.904; 0.04)  immunoglobulin kappa constant (-2.554; 0.04)  interleukin 1 receptor like 1 (2.362; 0.03  mitogen-activated protein kinase 8 (0.874; 0.031)  transforming growth factor beta 2 (1.276; 0.047)  Other:  mitogen-activated protein kinase 6 (0.676; 0.038)  **Predicted to be inhibited in participants who cleared the infection:**  Other:  Follistatin (3.388; 0.029)  lipase E, hormone sensitive type (1.122; 0.04)  SMAD family member 7 (0.57; 0.031) |

^a^The listed transcripts within the canonical pathway were identified using IPA, listed in ascending order by p-value, and sorted according to their predicted activation state and role. The fold change, listed first in each parenthetical expression, followed by the p-value, listed second, were determined by DESeq. IPA predicts gene activation or inhibition using the z-score, which is calculated by comparing the actual direction of gene expression in the experimental dataset (derived from log fold change) to the expected direction or pattern of gene expression (based on literature) when an entity is predicted to be activated [1].

Color coding: Red= role in oncogenesis; Blue= role in immune system; Green= role in epithelial barrier or cytoskeleton integrity.

[1] Krämer A, Green J, Pollard J, Tugendreich S. Causal analysis approaches in Ingenuity Pathway Analysis. Bioinformatics 2014;30:523–30. https://doi.org/10.1093/bioinformatics/btt703.
