# Supplementary material for: Schistosoma haematobium infection is associated with oncogenic gene expression in Cervical Mucosa, with enhanced effects following treatment: A pilot study
Source: PLoS Negl Trop Dis. 2025 Nov 21;19(11):e0013569. doi: 10.1371/journal.pntd.0013569 (PMC12637897; doi:10.1371/journal.pntd.0013569)
Supplement: S4 Table — (DOCX) [file pntd.0013569.s005.docx]

**Supplemental Table 4**. Top 9 differentially expressed genes between women with parasitological clearance post-praziquantel versus women without *S. haematobium* infection at baseline.

| **Gene name** | **NCBI**  **Gene ID** | **Gene function** | **Disease associations and references** | **Log2 Fold Change** |
| --- | --- | --- | --- | --- |
| Bone morphogenetic protein 2 | BMP2 | Development of bone and cartilage | Skeletal abnormalities and osteoporosis [1] | 1.7, p=8.70 x 10^-5^ by DESeq2, 1.2,  p=9.84 x 10^-4^ by Limma |
| C-X-C motif chemokine ligand 14 | CXCL14 | Encodes secreted proteins involved in immunoregulatory and inflammatory processes | Downregulated in HPV-positive head/neck and cervical cancers [2] | -2.2, p=4.72 x 10^-7^ by DESeq2, -1.6, p=9.44 x 10^-4^ by Limma |
| ENSG00000280149 (uncategorized gene) | N/A | N/A | N/A | -1.2 p=7.55 x 10 ^-5^  by DESeq2, -1.2, p=2.64 x 10^-4^ by Limma |
| Interleukin 1 receptor like 1 | IL1RL1 | Induced by proinflammatory stimuli, and involved in the function of helper T cells | Breast cancer [3] | 2.4, p=1.11 x 10^-4^  by DESeq2, 2.2, p=4.12 x 10^-4^ by Limma |
| Kinase insert domain receptor | KDR | Bone and cartilage development | Non-small cell lung cancer [4] and pregnancy complications [5] | 1.8 p=4.49 x 10^-5^  by DESeq2, 1.8, p=1.56 x 10^-5^  by Limma |
| Nuclear receptor subfamily 1 group D member 1 | NR1D1 | Negatively regulates core clock protein expression and may also modulate genes involved in metabolic, inflammatory, and cardiovascular processes | Bladder cancer [6] | 2.1 p=1.32 x 10^-5^  by DESeq2, 1.7, p=2.35 x 10^-4^ by Limma |
| Prolyl 3-hydroxylase 2 | P3H2 | Collagen chain assembly, stability and cross-linking | Breast cancer [7] | 1.6, p=1.88 x 10^-4^  by DESeq2, 1.4, p=3.25 x 10^-4^ by Limma |
| PRDM16 divergent transcript | PRDM16-DT/ LINC00982 | Controls bidirectional cell fate switch between skeletal myoblasts and brown adipocytes. | Breast [8] and renal cancer [9] | -1.7, p=7.58 x 10^-5^  by DESeq2, -1.5, p=8.56 x 10^-4^ by Limma |
| Rap guanine nucleotide exchange factor 5 | RAPGEF5 | GTPases function in signal transduction as GTP/GDP-regulated switches that cycle between inactive GDP- and active GTP-bound states | Renal [10] and bladder cancer [11] | 1.4, p=6.44 x 10^-5^  by DESeq2, 1.2, p=1.88 x 10^-4^ by Limma |

[1] Priestley JRC, Deshwar AR, Murthy H, D’Agostino MD, Dupuis L, Gangaram B, et al. Monoallelic loss-of-function BMP2 variants result in BMP2-related skeletal dysplasia spectrum. Genet Med 2023;25:100863. <https://doi.org/10.1016/j.gim.2023.100863>.

[] Cicchini L, Westrich J, Xu T, Vermeer D, Berger J, Clambey E, et al. Suppression of Antitumor Immune Responses by Human Papillomavirus through Epigenetic Downregulation of CXCL14. mBio 2016;7:e00270-16. https://doi.org/10.1128/mBio.00270-16.

[3] Sarmiento-Castro A, Caamaño-Gutiérrez E, Sims A, Hull N, James M, Santiago-Gómez A, et al. Increased Expression of Interleukin-1 Receptor Characterizes Anti-estrogen-Resistant ALDH+ Breast Cancer Stem Cells. Stem Cell Reports 2020;15:307–16. https://doi.org/10.1016/j.stemcr.2020.06.020.

[4] Kaira K, Imai H, Kawasaki T, Hashimoto K, Miura Y, Shiono A, et al. Potential of VEGFR2 expression as a predictive marker of PD-1 blockade in patients with advanced NSCLC. Oncol Rep 2022;48:214. https://doi.org/10.3892/or.2022.8429.

[5] Ho S, Chaput D, Sinkey R, Garces A, New E, Okuka M, et al. Proteomic studies of VEGFR2 in human placentas reveal protein associations with preeclampsia, diabetes, gravidity, and labor. Cell Commun Signal 2024;22:221. https://doi.org/10.1186/s12964-024-01567-0.

[6] Yang Y, Bai Y, Wang X, Guo Y, Yu Z, Feng D, et al. Clock gene NR1D1 might be a novel target for the treatment of bladder cancer. Urol Oncol 2023;41:327.e9-327.e18. https://doi.org/10.1016/j.urolonc.2023.04.021.

[7] Shah R, Smith P, Purdie C, Quinlan P, Baker L, Aman P, et al. The prolyl 3-hydroxylases P3H2 and P3H3 are novel targets for epigenetic silencing in breast cancer. Br J Cancer 2009;100:1687–96. https://doi.org/10.1038/sj.bjc.6605042.

[8] Chi F, Qiu F, Jin X, Chen L, He G, Han S. LINC00982 Inhibits the Proliferation, Migration, and Invasion of Breast Cancer Cells Through the miR-765/DPF3 Axis. DNA Cell Biol 2022;41:424–36. https://doi.org/10.1089/dna.2021.0866.

[9] Zhang C, Li X, Luo Z, Wu T, Hu H. Upregulation of LINC00982 inhibits cell proliferation and promotes cell apoptosis by regulating the activity of PI3K/AKT signaling pathway in renal cancer. Eur Rev Med Pharmacol Sci 2019;23:1443–50. https://doi.org/10.26355/eurrev_201902_17101.

[10] Chen Q, Liu T, Bao Y, Zhao T, Wang J, Wang H, et al. CircRNA cRAPGEF5 inhibits the growth and metastasis of renal cell carcinoma via the miR-27a-3p/TXNIP pathway. Cancer Lett 2020;469:68–77. https://doi.org/10.1016/j.canlet.2019.10.017.

[11] Wang C, Yang X. CircRAPGEF5 sponges miR-582-3p and targets KIF3A to regulate bladder cancer cell proliferation, migration and invasion. Int Immunopharmacol 2024;131:111613. https://doi.org/10.1016/j.intimp.2024.111613.
