# Supplementary material for: Schistosoma haematobium infection is associated with oncogenic gene expression in Cervical Mucosa, with enhanced effects following treatment: A pilot study
Source: PLoS Negl Trop Dis. 2025 Nov 21;19(11):e0013569. doi: 10.1371/journal.pntd.0013569 (PMC12637897; doi:10.1371/journal.pntd.0013569)
Supplement: Attachment — Submitted filename: DNA Tz FGS.pdf [file pntd.0013569.s007.pdf]

PNTD-D-24-0141

General comments:

It is a small sample after a logistically difficult study, there are some lessons in that, and transparency is good. However, it is very interesting and well-written manuscript, opening for other studies that could be groundbreaking.
The title is misleading:

1) The gene expression is measured in cervical mucosa whereas clearance of infection is measured systemically only.
2) As far as I can see no enhanced effects of treatment are described anywhere. This should be showcased in the discussion and alluded to in the introduction. Your MM should make it possible for the reader to see that this is in fact being measured

In the material and methods you need to define a number of terms, at least the following:

- vaginal cleansing
- Cleared infection (not a good term, ambiguous, see below). Please rephrase
- Predicted to be activated
- anticipated to be suppressed
- differentially expressed
- canonical

Specific comments

Line 86 genital alterations cannot "suggest". Please rephrase
Reference 15 does not provide original research on squamous cell carcinoma; I would suggest you pull out the best original paper on this. The evidence is weak
Line 158. Was the clinician blinded to the *S. haematobium* lab results. What kind of training did the clinician have in FGS lesion recognition?
Line 175 "Women who had schistosome infections received observed treatment with praziquantel (40 mg/kg) each time they were found to have infection" – as determined by which test(s)?
Figure 1. Please add in the follow-ups as the "final analysis", not baseline
Line 234. Rephrase "cleared infection". Alternatively define this. There are long-term consequences of S. haematobium that seem to last after the worms are dead,
Line 244 "differentially expressed" is this any difference or is it significant? Please define this term and the rationale for using it. In absolutely any dataset there will be data that is differentially expressed.
Line 245. Why IPA?

Results

1. The p-values are expressed in a non-conform way, please revert to p<0.001 – any more zeros are hard to read and clinically irrelevant. Please be clear and transparent, e.g. non-significant findings should not be shrouded in a lot of words.
2. Please, consistently, when comparing two phenomena, mention baseline first,

Table 1. At baseline the sandy patches are the same in the two groups… and very few. This should be discussed.
Table 1. The denominator in row 1 is confusing (n=20/39) and you have explained the exclusions in the text. Please remove "/39".
Table 1. The above point is also confusing for the data, one has to calculate if the results are a based on the 39 cases or only one the 20.
Table 1. Contraceptive use: were these hormonal contraceptives? Please clarify.
Table 1. Please define vaginal cleansing
Table 1. The blood vessels have been found to not be "new" (Jourdan et al) Therefore the correct term is "Abnormal blood vessels", not neovascularization

Table 1. I note that sandy patches were found in women without any positive lab analyses. I would suggest excluding them.
Line 300: “13 women were eligible 300 for continued follow up” Please mention reason here for further exclusions, please also add this into the Figure 1.
Line 319 Did you do STI analyses at all visits? Please clarify here if follow-up infections were found
Table 3. Why these particular analyses? What was your rationale.
Table 3. ENSG00000280149. Please, delete this row or explain why it’s there
Tables 2 and 3 are complementary. Table 3 belongs in the MM but pedagogically it is better situated where it is, I suggest to leave it here.
Line 354. “…cleared *S. haematobium* after praziquantel treatment versus women with *S. haematobium* infection…”. In other diseases, the term “infection” is usually not ambiguous. There are long-term consequences of *S. haematobium* beyond having urinary egg excretion/positive CAA but the studies on this have been done in endemic areas. Uninformed readers will think that the cervicovaginal lesions are gone when CAA/microscopy are negative. Therefore, the term “cleared infection” should replaced with precise terms: Negative urine microscopy/CAA (indicating worm death).
Line 355/Table 4. Define canonical in this scenario
Table 4 Rows 2 and onwards. Please explain what these figures mean, are they from the literature or study results, if study results, say which test:
2 (1.86; 0.000000503)
8 (1.014; 0.0000129)
1 (2.237; 0.000292)
(0.839; 0.0000784)
(0.881; 0.000228)
Line 340 and 376. What is the unit of the cut-off 0.05 xxx or is it a p-value?
If this is a p-value then this use should be eloborated under statistics
Line 361. P-values are not depicted in this table, where are they presented?
Lines 367-372/191-400. are these p-values? If yes, please use ordinary format or simply write “(not significant)“/p<0.001..
Line 373. Descending order of what
Lines 410 and below. This section seems to belong in the discussion – if not, please refer to figures/tables.

Discussion general comments

You are “listing” your findings and saying what they mean in other settings. However, you need to make inferences and really discuss the potential implications of having schistosomiasis

1) The direction of the associations is almost not discussed at all, making it very hard to see implications. We understand this field is in development, but interpretations should be expressed, albeit carefully. What are the implications for treatment – some of the factors seem to get worse - the “direction” of the findings should be clearer.
2) What could the implications for treatment
3) The gene expression is measured in cervical mucosa whereas clearance of infection is measured systemically only – how exactly do you see the biological connection?
4) You cannot really say anything about FGS or can you, please elaborate
5) Many sentences start with the lab results and end with who it affects – it’s better to reverse this.
6) I would suggest you divide the discussion in groups, one paragraph for each of the groups e.g. In women with patent systemic *S. haematobium* infection we found ..
7) Many long sentences, please shorten.

Line 429. – long sentence + the same content in following sentence. The first pragraph is important to catch the interest of the reader and should not be convoluted/repetitive. You are also just describing the methodology - not your own findings. In the Discussion chapter: Start with the most interesting findings, then your findings in perspective, then limitations, impact, recommendations, future research and future actions.
Line 435-438. Very long sentence that again says something on the methodology – and how unique this is - but nothing concrete You say how unique this is in lines 429 ("first"), 431 ("novel"), 434 ("not common") –once is enough
Line 442. Wrong reference
Line 448.

- Long sentence
- Does not say which direction the association was – did it get "better/worse.
- Start with who you are comparing (which group), then your finding. You want to first frame the reader's thoughts, then say what happened to them e.g.
  Comparing *S. haematobium* positive women with women who cleared urinary egg excretion/CAA, we found a decrease in genes associated with carcinogenesis, immune activation, inflammation, and epithelial integrity.

Line 451. Are these the same people as in the sentence before? If yes, start with e.g. Furthermore, as has been reported for women with ovarian tumours, ...
Start with who you are describing, then your finding
Line 453. This starts well but it is too long
Line 464. No reference to figures in Discussion
Lines 466 and the next sentences. Please look through every sentence.

- Long sentences
- Start with the women with disease/ at baseline, then other group, frame the reader's thoughts, then say what "happened" to them.

Line 478. more differences – in which direction? Was it worse or better?
Line 481. Move to MM: We defined clearance of *S. haematobium* as the reduction of CAA levels post-treatment, which indicates a successful eradication of live adult worms that secrete this molecule into the bloodstream.
Line 487. Kallestrup et al found the opposite in Zimbabwe
Line 489. Altered – in which direction? Which genes, be concrete.

References:

Ref 3. This cannot be right – spellings, place, etc: https://www.who.int/teams/control-of-neglected-tropical-diseases/schistosomiasis/epidemiology
Ref 7. Petry is very old and a not a good study. There are several papers after 2010 that discuss HPV, please use those. E.g. you already have ref 69
Ref 12. Should be replaced by https://pubmed.ncbi.nlm.nih.gov/16406034/
Ref 13. Rather use this one: https://www.frontiersin.org/journals/tropical-diseases/articles/10.3389/fitd.2024.1322652/full
the current Ref constitutes a handful of cases, and the baseline and follow-up investigators were not the same person
Ref 89. This paper does not explore cancer
REF 90 + 91. Again – do a literature dive in the last years papers on schisto and ca. cervix/HPV
