# Supplementary material for: Schistosoma haematobium infection is associated with oncogenic gene expression in Cervical Mucosa, with enhanced effects following treatment: A pilot study
Source: PLoS Negl Trop Dis. 2025 Nov 21;19(11):e0013569. doi: 10.1371/journal.pntd.0013569 (PMC12637897; doi:10.1371/journal.pntd.0013569)
Supplement: Attachment — Submitted filename: PLOS NTD Reviewer Responses.docx [file pntd.0013569.s008.docx]

**Responses to Reviewer`s comments**

**Reviewer 1**

**1. Very minor typo: Lines 91-92 “In S. haematobium-associated bladder cancer, S. haematobium eggs are classified as Group 1 carcinogen” should be “a Group 1 carcinogen”**

We thank the reviewer for the thorough observation. We corrected the typo.

**2. This is a very interesting manuscript but I do have methodological concerns:
24 samples failed to pass QC for RNA-Seq and only 39 passed. Likewise, many women (7) were not eligible for continued follow up after praziquantel treatment. The authors should better emphasize these important limitations**
We thank the reviewer for this important observation and the suggestion. As suggested, we now mention this in the limitations of our study to ensure proper transparency within the discussion (lines 597- 599).

**3. The lack of significant pathways (using IPA) comparing women with and without Sh is concerning. The authors should consider analyzing their data by modifying their pipeline (i.e., GSEA).**

We agree with the reviewer’s comment and that the lack of significant pathways between women with and without Sh was not expected given that there were differentially expressed genes observed. To confirm this finding, in addition to using Ingenuity Pathway Analysis (IPA), we also analyzed our results using a second, independent pipeline (Webgestalt Kyto Encyclopedia of Genes and Genomes, KEGG). IPA is a proprietary program that uses a well-curated and reliable database to investigate potential biological pathways, networks, and disease associations using pathway enrichment analysis, upstream regulator prediction, and gene interaction mapping. KEGG operates differently, by emphasizing canonical pathways and open-access data, and unlike IPA, does not integrate experimental evidence for upstream-downstream relationships. Together, they provide a deeper understanding of both known pathways and emergent regulatory mechanisms. These two distinct and independent analytic tools strengthen our conclusion that, at least in this small pilot study, no altered pathways are identifiable between these two groups. It is possible that this is due to our small sample size.

**4. The genes involved in oncogenesis in Table 4 – what is the direction of differential gene expression for women who cleared infection post-praziquantel vs. women with baseline Sh?**

Thank you for your comment. As indicated in now Supplemental table 3 (former table 4), the subheadings in the last column specify the predicted direction of the genes. The subheadings state, “Predicted to be activated in participants who cleared the infection,” and “Predicted to be inhibited in participants who cleared the infection.”

**Reviewer 2**

**The title is misleading:**

We thank the reviewer for this important comment. We have now revised and clarified the title to be: *“Schistosoma haematobium* Infection is associated with Oncogenic Gene Expression in Cervical Mucosa, with Enhanced Effects Following Treatment.”

1) **The gene expression is measured in cervical mucosa whereas clearance of infection is measured systemically only.**

We thank the reviewer for this insightful and important comment. The reviewer is correct that we defined clearance of infection systemically, by CAA negativity, as well as by the absence of urine eggs. We did not have the capacity to assess for actual presence or clearance of eggs within the genital tract. For clarity, we added a figure (Fig. 3) showing the different group comparisons, as well the diagnostic tests by which the groups were defined. We hope this will improve the clarity of our manuscript.

2) **As far as I can see no enhanced effects of treatment are described anywhere. This should be showcased in the discussion and alluded to in the introduction. Your MM should make it possible for the reader to see that this is in fact being measured.**

Thank you for your valuable comment. The new figure that we have added will hopefully help to clarify this, with Comparisons #2 and #3 labeled, “Post-Treatment.” We have also revised sections of the introduction and discussion to highlight this point further.

3) **In the material and methods you need to define a number of terms, at least the following:**

**- vaginal cleansing**

**- Cleared infection (not a good term, ambiguous, see below). Please rephrase.**

**- Predicted to be activated**

**- anticipated to be suppressed**

**- differentially expressed**

**- canonical**

We thank the reviewer for this insightful comment. To improve clarity and ensure consistent understanding, we have included the following definitions in the Materials and Methods section:

- **vaginal cleansing:** In Tanzania, vaginal cleansing practices are common and vary widely in method and purpose. Women often use water alone for cleansing, but traditional or commercial products are also employed. Cleansing methods include inserting substances such as herbs, honey, or commercial products like detergents, jelly, or medications, typically applied with fingers or occasionally with cloths. These practices may occur for hygiene purposes, in relation to menstruation, or as part of sexual preparation or aftercare. Most cleansing is done intravaginally, and it is often culturally influenced. To support this, we added the citation by Francis et al, *Sex Transm Dis*, 2013.
- **Cleared infection (not a good term, ambiguous, see below). Please rephrase:** We appreciate this very helpful suggestion both here and below. We have changed the terminology from “cleared infection” to “parasitological clearance post-praziquantel treatment” and defined that this term reflects the measured absence of *S. haematobium* eggs in urine as well as negative serum CAA in figure 3.
- **Predicted to be activated**: Refers to molecular pathways, biological processes, or upstream regulators that are inferred by IPA to have increased activity based on the pattern of input gene expression data. This prediction is made using the z-score algorithm, where a positive z-score indicates activation.
- **Predicted to be inhibited**: We have changed this term to “predicted to be inhibited” so that it is more clearly the opposite of “predicted to be activated.” This term refers to molecular pathways, biological processes, or upstream regulators that are inferred by IPA to have decreased activity based on the pattern of input gene expression data. A negative z-score indicates suppression.
- **Differentially expressed**: Refers to genes that exhibit statistically significant differences in copy numbers of genes expressed between the experimental and control conditions. These differences are quantified by comparing gene copy numbers for statistically significant differences, or fold changes, between two groups after adjusting for multiple comparisons. A threshold for a meaningful fold change of >2 or <−2 was also applied.
- **Canonical**: Refers to well-established, curated signaling or metabolic pathways that are broadly recognized in the scientific literature. IPA uses these canonical pathways to provide a framework for interpreting gene expression data and identifying pathway-level changes.

These definitions have been added to the Materials and Methods section under the heading “Terminology” starting in line 272 as well as below table 1 under the asterisk “b” to address the reviewer’s concerns and ensure clarity for readers.

**Line 86 genital alterations cannot “suggest”. Please rephrase**

We thank the reviewer for their input and have rephrased this from “suggest” to “may uncover.”

**Reference 15 does not provide original research on squamous cell carcinoma; I would suggest you pull out the best original paper on this. The evidence is weak**

We appreciate the reviewer’s suggestion and have added the original publication by Bernardo et al (reference #16) as a recent original research reference. We have kept the reference for systematic review as well, as it provides a comprehensive overview including this Bernardo study plus the other studies investigating S. haematobium in patients with bladder cancer.

**Line 158. Was the clinician blinded to the *S. haematobium* lab results. What kind of training did the clinician have in FGS lesion recognition?**

Thank you for your insightful comments. To ensure consistency and accuracy in diagnosing FGS, the entire study team was trained in FGS recognition and evaluated lesions in comparison to the WHO FGS Atlas as a reference during examinations. No colposcope was used during examinations. All findings were reviewed and agreed upon by at least two study team members to maintain diagnostic reliability. Study team members were not blinded to *S. haematobium* infection status. We have clarified this process in the manuscript for better transparency in 174- 175 and line 186.

**Line 175 “Women who had schistosome infections received observed treatment with praziquantel (40 mg/kg) each time they were found to have infection” – as determined by which test(s)?**

Thank you for requesting this point of clarification. Women were treated any time were found to be infected with *S. haematobium* by either positive urine microscopy or serum CAA ≥30 pg/mL. We have now specified this. Further, we included this definition of “infected” in the Materials and Methods section to clarify that the term "infected" at any time point refers to positive urine microscopy and/or serum CAA ≥30 pg/mL. Additionally, as mentioned earlier, we added figure 3 to provide a clearer overview of the groups and the tests used to define them.

**Figure 1. Please add in the follow-ups as the “final analysis”, not baseline.**

Thank you for this helpful suggestion. We altered figure 1 to also include the women at follow up and listed reasons for exclusions.

**Line 234. Rephrase “cleared infection”. Alternatively define this. There are long-term consequences of S. haematobium that seem to last after the worms are dead,**

We agree with the reviewer and are grateful for this suggestion. As mentioned above we changed the term to “women with parasitological clearance post-praziquantel” and defined the term in the methods section, as well as in the newly added figure 3.

**Line 242 “differentially expressed” is this any difference or is it significant? Please define this term and the rationale for using it. In absolutely any dataset there will be data that is differentially expressed.**

We thank the reviewer for this important point. We inadvertently omitted the word “significantly” in this sentence and are only seeking features of the dataset that are statistically (and biologically) significant. We have revised the text in line 265 so that it now reads **“**All genes identified as being significantly differentially expressed with a Padj of ≤0.05.”

**Line 245. Why IPA?**

We appreciate this question. We used both IPA and KEGG in order to increase our data’s robustness, since the two analytical tools work differently. We have provided more detail on this in the above response to the 3^rd^ question of Reviewer 1.

**Results**

1. **The p-values are expressed in a non-conform way, please revert to p<0.001 – any more zeros are hard to read and clinically irrelevant. Please be clear and transparent, e.g. non-significant findings should not be shrouded in a lot of words.**
2. We thank the reviewer for this suggestion. After reviewing other similar manuscripts and conventions in the field, we believe it important to list the actual p-values and have kept them as they are. Providing the actual p-values in our manuscript enhances transparency, allowing readers to critically assess the significance of the findings (Aguinis et al *BMJ Evid Based Med*. 2019). It also supports reproducibility, as detailed values enable others to replicate the analysis. Additionally, exact values provide more context about the strength of the statistical evidence, offering a nuanced understanding that aids in the interpretation of results and comparison across studies. We believe this level of detail ultimately strengthens the credibility and scientific rigor of our manuscript.
3. **Please, consistently, when comparing two phenomena, mention baseline first,**

Thank you for your feedback. However, we believe the current phrasing in our results section is clear and consistent with standard conventions. Here, we are consistently placing the reference group last in comparisons, though sometimes the reference group is people at baseline compared to those post-treatment. Therefore, the current structure in the results section accurately conveys the intended meaning. Nevertheless, we did change the order whenever sensible in the section of the discussion as suggested below.

**Table 1. At baseline the sandy patches are the same in the two groups… and very few. This should be discussed.**

We appreciate the insightful comment. As suggested, we addressed this now in the discussion in a paragraph dedicated to FGS on why we chose not to restrict our cohort of women to solely confirmed genital *S. haematobium* infection (lines 569- 578).

**Table 1. The denominator in row 1 is confusing (n=20/39) and you have explained the exclusions in the text. Please remove ”/39”.**

**Table 1. The above point is also confusing for the data, one has to calculate if the results are a based on the 39 cases or only one the 20.**

Thank you for raising this point. We agree with the reviewer and removed the denominator in row 1 as suggested.

**Table 1. Contraceptive use: were these hormonal contraceptives? Please clarify.**

We agree with the suggestion and clarified which contraceptives were included. An asterisk “a” was added defining the possible modes of contraceptives which were used under table 1.

**Table 1. Please define vaginal cleansing**

We agree that a definition is needed. We have now added a definition and further clarification in the footnote of table 1.

**Table 1. The blood vessels have been found to not be “new” (Jourdan et al) Therefore the correct term is “Abnormal blood vessels”, not neovascularization**

We thank the reviewer for this helpful suggestion. We changed the term to “Abnormal blood vessels,” as suggested by the reviewer.

**Table 1** **I note that sandy patches were found in women without any positive lab analyses. I would suggest excluding them.**

Sandy patches on the cervix, commonly associated with FGS, have an approximate sensitivity of 81% and only a specificity of 42% according to Galappaththi-Arachchige et al, *PLoS One* 2018.

Sandy patches may also persist in women with *S. haematobium* infection following treatment (Kjetland et *al Trans R Soc Trop Med Hyg.* 2006). For these reasons, sandy patches are not pathognomonic for FGS and we therefore chose not to exclude these women. We have now discussed this more thoroughly in the discussion section, including the possibility that some of the uninfected women had these sandy patches possibly from prior infection or from concurrent FGS despite negative CAA and urine microscopy.

**Line 300: “13 women were eligible 300 for continued follow up” Please mention reason here for further exclusions, please also add this into the Figure 1.**

We appreciate this suggestion. We have now altered figure 1 to include women who had follow up samples available and the reasons for excluding women at follow up.

**Line 319 Did you do STI analyses at all visits? Please clarify here if follow-up infections were found**

We appreciate the reviewer’s comment and have added the requested information to the manuscript. Yes, we did STI analyses at all visits. Specifically, we now detail that five STIs were diagnosed at follow-up, including three cases of trichomonas infections (two at the 6-month visit and one at both the 6- and 12-month visits) and two cases of gonorrhea (one at the 4-month visit and one at the 6-month visit). This information has been incorporated into the results and methods sections for clarity.

**Table 3. Why these particular analyses? What was your rationale.**

The reason for these analyses was the low number of samples. We have described this at the beginning of the results section (lines 358- 367), where we write: “Due to the low number of samples, a paired analysis could not be performed for women with persistent infections and comparisons between different months post-treatment could not be made. We therefore performed three comparisons, each of which is described in detail in the following sections: (1) gene expression in women with versus without *S. haematobium* infection at baseline; (2) gene expression in women with parasitological clearance post-praziquantel treatment versus women with baseline *S. haematobium* infection; and (3) gene expression in women with parasitological clearance post-praziquantel treatment versus women without *S. haematobium* infection at baseline (Figure 3).”

**Table 3. ENSG00000280149. Please, delete this row or explain why it’s there**

We have kept these novel genes in the table because they were among the top genes found to be significantly differently expressed. Although at present no data is available on their function, it is possible that the function of these genes may be defined in the future and that they then could provide additional valuable insights into *S. haematobium* gene expression in the genital tract.

**Tables 2 and 3 are complementary. Table 3 belongs in the MM but pedagogically it is better situated where it is, I suggest to leave it here.**

We thank the reviewer for mentioning this. After consulting with Dr. Bishoy Faltas, a highly experienced oncologist with extensive expertise in molecular studies at our institution, we decided to move tables 2 through 5 (now supplemental table 1 through 4) to the supplementary material.

**Line 354. “…cleared *S. haematobium* after praziquantel treatment versus women with *S. haematobium* infection…”. In other diseases, the term “infection” is usually not ambiguous. There are long-term consequences of *S. haematobium* beyond having urinary egg excretion/positive CAA but the studies on this have been done in endemic areas. Uninformed readers will think that the cervicovaginal lesions are gone when CAA/microscopy are negative. Therefore, the term “cleared infection” should replaced with precise terms: Negative urine microscopy/CAA (indicating worm death).**

Thank you for his valuable comment. We completely agree and have changed the terminology as suggested above throughout the manuscript to ensure clarity.

**Line 355/Table 4. Define canonical in this scenario**

Canonical pathways, as defined in IPA, are well-established, curated biological pathways that represent fundamental and widely accepted molecular and cellular processes. These pathways are derived from peer-reviewed literature and serve as standardized models for analyzing experimental data by mapping gene or protein interactions within known biological networks. We have now added this definition to the Methods section under “Terminology” in lines 270- 284.

**Table 4 Rows 2 and onwards. Please explain what these figures mean, are they from the literature or study results, if study results, say which test:**

**2 (1.86; 0.000000503)**

**8 (1.014; 0.0000129)**

**1 (2.237; 0.000292)**

**(0.839; 0.0000784)**

**(0.881; 0.000228)**

Thank you for pointing out the need for further clarification. To make this clearer, we now list the syntax of the parenthetical expression at the top of the right-hand column and we describe the statistical test used and the meaning of the expression beneath table 4. As described in the Methods section, these are our study results, for which DESeq was used to analyze the transcriptome results and used for IPA and KEGG pathway analysis.

**Line 340 and 376. What is the unit of the cut-off 0.05 xxx or is it a p-value?**

**If this is a p-value then this use should be elaborated under statistics**

We thank the reviewer for this comment. This cut-off of 0.05 is the false discovery rate that we allowed for this analysis. The false discovery rate adjusts for the problem of multiple comparisons to ensure overall reliability in the results. We have clarified this in the Methods (lines 259- 264).

**Line 361. P-values are not depicted in this table, where are they presented?**

We thank you for pointing this out. We have added the p-values to the tables.

**Lines 367-372/191-400. are these p-values? If yes, please use ordinary format or simply write “(not significant)“/p<0.001..**

Please see the above answer in regards to listing detailed p-values and why we have kept these very small values as the standard practice in presentations of RNA-Seq data.

**Line 373. Descending order of what**

We clarified that it is in the descending order of statistical significance in line 416.

**Lines 410 and below. This section seems to belong in the discussion – if not, please refer to figures/tables.**

We thank the reviewer for this comment. We apologize, as we did not mention that this section belongs to figure 7 (formerly figure 6). We adjusted the manuscript accordingly in lines 462 and 465. Additionally, we shortened the entire paragraph for better readability.

**Discussion general comments**

**You are “listing” your findings and saying what they mean in other settings. However, you need to make inferences and really discuss the potential implications of having schistosomiasis**

**1) The direction of the associations is almost not discussed at all, making it very hard to see implications. We understand this field is in development, but interpretations should be expressed, albeit carefully. What are the implications for treatment – some of the factors seem to get worse - the “direction” of the findings should be clearer.**

We thank the reviewer kindly for this insightful comment. We altered our discussion accordingly to discuss the possible implications of our findings further.

**2) What could the implications for treatment**

We thank the reviewer for this important feedback. We now address this pertinent implication in the discussion.

**3) The gene expression is measured in cervical mucosa whereas clearance of infection is measured systemically only – how exactly do you see the biological connection?**

We appreciate this question and have added a full paragraph in the discussion to address the fact that we were unable to diagnose FGS by parasite DNA or eggs in the genital tract. We further now discuss the possible reasons that, despite analyzing women grouped by the presence or absence of systemic infection, we identified significant differences in cervical mucosal gene expression.

**4) You cannot really say anything about FGS or can you, please elaborate**

Thank you for raising this important point. We agree that our original manuscript did not explicitly address FGS in sufficient detail. While our study does not directly assess FGS, the findings are relevant to understanding the broader immunological and clinical context of *S. haematobium* infections, which involve the genital tract in the majority of cases. In fact, the fact that we identified significant differences in gene expression in the genital tract in women enrolled based on confirmation of systemic *S. haematobium* infection only further underscores that this parasite does frequently affect the genital tract in women living with infection.

We have elaborated on the potential implications of our findings for FGS in the Discussion section as described in answer to the previous question (lines 559- 578). We also acknowledge the limitations of our study regarding direct conclusions about FGS and suggest this as an area for future investigation. We hope this addition clarifies the relevance of our study to FGS while appropriately contextualizing its limitations.

**5) Many sentences start with the lab results and end with who it affects – it’s better to reverse this.**

We appreciate this observation. As suggested, we reversed the order, starting with the group and ending with the result whenever sensible.

**6) I would suggest you divide the discussion in groups, one paragraph for each of the groups e.g. In women with patent systemic *S. haematobium* infection we found…** Thank you for this helpful suggestion. We have reorganized the discussion, separating it more by groups rather than themes, as recommended. **7) Many long sentences please shorten Line 429. – long sentence + the same content in following sentence. The first pragraph is important to catch the interest of the reader and should not be convoluted/repetitive. You are also just describing the methodology - not your own findings. In the Discussion chapter: Start with the most interesting findings, then your findings in perspective, then limitations, impact, recommendations, future research and future actions.**

We thank the reviewer for these comments. We shortened this sentence to only mention that our study builds on a prior cross-sectional study conducted in Tanzania and reworked the entire Discussion section.

**Line 435-438. Very long sentence that again says something on the methodology – and how unique this is - but nothing concrete You say how unique this is in lines 429 (“first”), 431 (“novel”), 434 (“not common”) –once is enough**

Thank you for this comment. We shortened the sentence to “Cervical transcriptomic studies thus far have linked altered gene expression solely to HPV, cervical cancer, or gynecological syndromes like endometriosis but not to parasitic infections.” We agree that once is enough and have now kept the word “first” but removed “novel” and “not common.”

**Line 442. Wrong reference Ref (69)**

Thank you for pointing this out. We have removed this reference.

**Line 448.**

**- Long sentence**

**- Does not say which direction the association was – did it get “better/worse.**

**- Start with who you are comparing (which group), then your finding. You want to first frame the reader’s thoughts, then say what happened to them e.g. Comparing *S. haematobium* positive women with women who cleared urinary egg excretion/CAA, we found a decrease in genes associated with carcinogenesis, immune activation, inflammation, and epithelial integrity.**

As suggested, we have rearranged the discussion, rewritten our sentences with the group first, and shortened sentences whenever possible.

**Line 451. Are these the same people as in the sentence before? If yes, start with e.g. Furthermore, as has been reported for women with ovarian tumours, …. Start with who you are describing, then your finding**

As suggested, we changed the sentence to, “In these same women, the top identified canonical pathway in women after treatment was “Sertoli cell-germ cell junction pathway,” which has also been described in women with ovarian tumors.”

**Line 453. This starts well but it is too long**

Thank you for this comment. We shortened the sentence to improve readability as, “Moreover, in these women, the IPA analysis anticipated inhibition of multiple genes (HNF4G, HNF4A, GMNN, SOX1, SOX3) that have been described to be downregulated in multiple types of cancer [60–65], including cervical cancer [66–69].”

**Line 464. No reference to figures in Discussion**

We appreciate this suggestion and added the references to the figures in lines 462 and 465.

**Lines 466 and the next sentences. Please look through every sentence.**

**- Long sentences**

**- Start with the women with disease/ at baseline, then other group, frame the reader’s thoughts, then say what “happened” to them.**

Thank you for this insightful comment. We have worked on and edited the entire discussion accordingly.

**Line 478. more differences – in which direction? Was it worse or better?**

Since women after treatment did worst, we restructured the sentence to emphasize this and make it clearer: “Instead, women with parasitological clearance post-praziquantel treatment, compared both to women with infection and to women without, we found more exhibited severer cervical transcriptomic changes that were associated with further increased oncogenic and inflammatory activity and reduced mucosal integrity.”

**Line 481. Move to MM: We defined clearance of *S. haematobium* as the reduction of CAA levels post-treatment, which indicates a successful eradication of live adult worms that secrete this molecule into the bloodstream.**

We moved this to the methods section as suggested (lines 160- 164).

**Line 487. Kallestrup et al found the opposite in Zimbabwe**

We appreciate the reviewer’s comment. Brown and colleagues reported a transient increase in HIV viral load at 1 month, which had normalized by 5 months, while Kallestrup and colleagues demonstrated a decrease in viral load by 3 months. However, these observations are not necessarily discordant because investigators measured post-treatment viral loads at different time points. Another way to interpret this would be that there is a transient worsening in that normalizes by 3 months (Kallestrup) and 5 months (Brown). We have now brought this point into the discussion and cite both Brown and Kallestrup (Reference number #74, line 563).

**Line 489. Altered – in which direction? Which genes, be concrete.**

Thank you for your request on clarification. We have decided to remove this sentence.

**References:**

We thank the reviewer for the careful review of the listed references. We have adjusted each as follows:

**Ref 3. This cannot be right – spellings, place, etc: https://www.who.int/teams/control-of-neglected-tropical-diseases/schistosomiasis/epidemiology**

We have removed this reference and adjusted the introduction accordingly.

**Ref 7. Petry is very old and a not a good study. There are several papers after 2010 that discuss HPV, please use those. E.g. you already have ref 69**

We agree with the reviewer. We have removed Reference #7, included the Kjetland reference (now reference #5), and added a new systematic review that was just published by Sturt and colleagues in *BMC Womens Health*.

**Ref 12. Should be replaced by** [**https://pubmed.ncbi.nlm.nih.gov/16406034/**](https://pubmed.ncbi.nlm.nih.gov/16406034/)

We replaced the reference as suggested.

**Ref 13. Rather use this one:** [**https://www.frontiersin.org/journals/tropical-diseases/articles/10.3389/fitd.2024.1322652/full**](https://www.frontiersin.org/journals/tropical-diseases/articles/10.3389/fitd.2024.1322652/full) **the current Ref constitutes a handful of cases, and the baseline and follow-up investigators were not the same person**

We replaced the reference as suggested.

**Ref 89. This paper does not explore cancer**

We removed this reference and apologize for our mistake.

**REF 90 + 91. Again – do a literature dive in the last years papers on schisto and ca. cervix/HPV**

We thank the reviewer for pointing this out. We added the study from Swai et al who found that only 25% of women infected with diagnosed *Schistosoma* had cervical cancer (reference #82). Additionally, we added the recently published study by Lamberti et al (reference #81) and the systematic review by Sturt et al.

**Additional corrections by the authors**

We noticed that we incorrectly mention 7 instead of the actual identified 9 genes in the abstract and author summary of our manuscript. We sincerely apologize for this mistake and corrected this in line 44.

We added the citation by Cicchini et al to table 2 for the gene CXCL14 to emphasize that its decrease has been reported in HPV-related cancers.
