# Supplementary material for: Schistosoma haematobium infection is associated with oncogenic gene expression in Cervical Mucosa, with enhanced effects following treatment: A pilot study
Source: PLoS Negl Trop Dis. 2025 Nov 21;19(11):e0013569. doi: 10.1371/journal.pntd.0013569 (PMC12637897; doi:10.1371/journal.pntd.0013569)
Supplement: Attachment — Submitted filename: Responses to Reviewes.docx [file pntd.0013569.s009.docx]

**Responses to Reviewers’ comments**

**General Comments**

1. Expert reviewers in the fields of schistosomiasis and infectious diseases have evaluated your revised manuscript. They appreciated the revisions you have made. However, one reviewer has suggested additional areas for improvement before the manuscript can be fully accepted by PNTD. In particular, validation of the nine genes identified as most differentially expressed in your RNA-seq analysis using RT-qPCR is essential to confirm the reliability of the observed expression patterns.

**We appreciate the efforts of Reviewers 1, 2, 3, and 4 and the editors in evaluating our manuscript.  We are happy to hear that the revisions that we have made were appreciated and we thank Reviewer 3 for the additional comments. We appreciate the request to confirm our findings via RT-qPCR and agree that doing so could further strengthen the findings of this pilot study. Unfortunately, we do not have additional material left from the cervical cytobrushes that could be used for PCR. Due to this limitation, we were as robust and unbiased as possible with our statistical process in selecting these genes by using both DESeq2 and Limma in R to identify the top nine consistently differentially expressed genes between two groups.**

**In past work on *S. haematobium,* we performed RNA-Seq in peripheral blood samples and in that work confirmed the observed changes in gene expression using qPCR (Dupnik KM et al, *Infect Immun* 2018, doi: 10.1128/IAI.00291-18). Of note, peripheral blood yields a far greater quantity of sample available for analysis than does cervical brushing, so this was more feasible for those samples. We further note that there is a growing consensus that results from RNA-Seq are expected to be reliable and that validation by qPCR is not always required (Coenye T, *Biofilm* 2021, doi: 10.1016/j.biofilm.2021.100043).**

**To address the reviewer’s concerns, we have now added this as a limitation in our discussion section (lines 573-574). Here and below, we cite line numbers as shown on the “Track Changes” version of the manuscript. We also have clarified in our title, abstract, and discussion that this is pilot, exploratory work.**

2. It is also important to discuss whether cervical cancer in this context may be related to somatic mutations in genes induced by the presence of *S. haematobium*, and/or whether it is associated with inflammation, fibrosis, and alterations to the epithelial cell microenvironment caused by the presence of *S. haematobium* eggs. These are valid scientific questions that need to be addressed.

**We agree with and appreciate these comments. We have now added both of these possibilities, together with additional references, to the discussion section (lines 528, 514-518).**

3. Finally, please ensure that all data used in this manuscript are publicly available, with a functional link to the data repository included in the manuscript. Sequence data should be submitted to the SRA repository.

**We thank the editor for this comment. Our approved and signed NIH Genomic Data Sharing plan permits sharing of gene-level summary data (gene names and copy numbers), but not raw RNA-Seq reads. Although the consent supports broader data sharing, we are currently bound by the limitations of our GDS plan. Depositing the reads would require us to revise our GDS plan and obtain new written NIH approval for a change in the NIH grant awarded. Instead, we are happy to provide the summary-level data in compliance with our approved plan.**

Reviewer 3 Comments:

1. Objectives are clear, the study design is apropriate, the population appropriate, the sample size is small, statistical analysis is correct, ethical and regulatory requirements seems to be well done.

**We thank the reviewer for these positive comments and agree that the sample size is somewhat small. However, for exploratory studies of gene expression, this is not an atypical size and we note that, even with this sample size, we were able to detect significant differences in gene expression.**

2. Conclusions are not supported by data, and they need to be clear. Authors discussion is not helpful to advance our understanding of this topic.

**We have now worked to strengthen our discussion section to bring clarity, following the additional comments outlined below, and we respond to each in more detail below.**

3. Authors have choice an interesting, relevant and pertinent scientific topic. This is a strong work developed by scientists with domain of conceptual and instrumental tools, medical and scientific. They need to be stimulated to go on working on this scientific topic: urogenital schistosomiasis. However, results described in the manuscript does not sustain authors’ initial proposal. Small amount of patients? Associated infections? Controversial strategy? Authors studding cytobrush samples state “altered cervical mucosal gene expression with oncogenic potential in patients infected with S. haematobium”. What this means?

**We appreciate this comment and agree with the reviewer that the small number of patients and the complexity of other associated infections complicates our analysis. We have added to the title, abstract, introduction, and discussion sections that this is a pilot study. We reported abundant sociodemographic and clinical data, none of which indicate statistically significant differences between women with versus without *S. haematobium* infection, but we recognize that this could be due to sample size. We have now addressed this more thoroughly in the limitations section (lines 569-582).**

**We have also reworded the statement about “Women with *S. haematobium* infection and those with recent parasitological clearance had altered cervical mucosal gene expression with oncogenic potential*.”* We now have revised this so that it reads, “Women with *S. haematobium* infection and those with recent parasitological clearance were found to have cervical gene alterations which have been reported in various cancers" (abstract lines 52-54).**

4. Statistic relevance derived from a p value <0.05 for selected genes is not enough.

**We agree with the reviewer that a p value of <0.05 would not be correct, and we did not use p-values of <0.05 in this manuscript. This is stated in the Methods section (lines 259-261) where we write, “Corrected P values were calculated using the Benjamini-Hochberg method adjustment for multiple testing to control the false discovery rate at ≤0.05.”**

5. The nine genes, selected, and expressed differentially should be validated with another test, for instance, Real Time PCR. Because selected patients are too complex with associated infections.

**We appreciate this comment and sincerely wish that we could validate these genes using real-time PCR. Unfortunately, the quantities of RNA collected by cervical cytobrush are small and we do not have any cytobrush sample left that could be used for this purpose. We do note that, in past work in which we performed RNA-Seq in peripheral blood in *S. haematobium* infection and the sample quantity was greater, we did validate our RNA-Seq findings by qPCR in a very similar population (Dupnik KM et al, *Infect Immun* 2018, doi:** [**10.1128/IAI.00291-18**](https://doi.org/10.1128/iai.00291-18)**).**

**We agree that other associated factors could affect our results, but we note that the frequencies of other genital infections in women with versus without *S. haematobium* infection were similar, as were sociodemographic characteristics (Table 1).  None of the differences reached statistical significance. The design and conduct of future studies that are larger in size to account for co-infections and that will include collection of additional samples for validation are goals for our team as we continue to work in this area.**

**To reflect these complexities, we have decided to reword the title, abstract, introduction and discussion to emphasize that this is a pilot project, and we have added our inability to perform confirmatory real-time PCR as a limitation (lines 572-576).**

6. “Women with S. haematobium infections compared to uninfected women, had gene expression consistent with enhanced oncogenesis in genital mucosa”. This is not clear to me and need to be proved, I am afraid.

**We thank the reviewer for this comment and have reworded this statement so that it is not so strong. We now write, “We found that women with *S. haematobium* infection compared to uninfected women, and even more that women who experienced parasitological clearance of infection compared to uninfected women, had altered genital tract expression of genes that have been associated with cancers” (lines 489- 492).**

7. And it is not clear also the reason to select LINC02084 gene to discuss. Maybe because is thought to be related with other cancers discussions.

**We thank the reviewer for this comment. We chose to highlight LINC02084 due to its differential expression and previously reported roles in epithelial tissue pathobiology, including in cancers of the urogenital tract, and past studies linking expression of this long intergenic non-coding RNA with proliferation and cancer progression. This is especially intriguing given that *S. haematobium* is a known cause of bladder cancer, and it is conceivable that it may also contribute to carcinogenesis in other urogenital compartments.**

8. Schistosoma haematobium is assumed as a carcinogenic pathogen. However, the mechanisms implicated in carcinogenesis are not yet clear. Cervical cancer related with S. haematobium infection is so important to be studied. The epistemology of this form of cancer is related with somatic mutations in genes or, on contrary, is related with inflammation, fibrosis and epithelial cells microenvironment alterations? In my point of view, somatic mutations are epiphenomena.

**We appreciate the reviewer’s thoughtful question and the suggestion that we discuss this in greater depth and have now done so (lines 514-518). We have added a discussion of what is known about the development of bladder cancer in *S. haematobium* infection, emphasizing the strong mechanistic data supporting the concept that inflammation and epithelial cell responses are early steps in tumor pathogenesis. Our pilot RNA-Seq data from the cervix are concordant with these findings, similarly reporting gene expression consistent with inflammation and cell proliferation. We have added further references to support this discussion (Bernardo C et al, *Tumor Biol* 2016; Nacif-Pimenta R et al, *Sci Rep* 2019; Santos LL et al, *J Clin Med* 2021).**

**We appreciate these helpful comments that have given us the opportunity to further strengthen our manuscript.**
